# Postliver Transplantation Rhino-Orbital Mucormycosis, an Unexpected Cause of a Downhill Course

**DOI:** 10.1155/2022/5413315

**Published:** 2022-05-28

**Authors:** Swapnali Sabhapandit, Mithun Sharma, Anuradha Sekaran, Balachandran Menon, Anand Kulkarni, Soumya TR, Padaki Nagaraja Rao, Duvurr Nageshwar Reddy

**Affiliations:** ^1^Department of Ophthalmology, AIG Hospitals, Hyderabad, India; ^2^Department of Hepatology and Liver Transplantation, Asian Institute of Gastroenterology, Hyderabad, India; ^3^Department of Pathology, Asian Institute of Gastroenterology, Hyderabad, India; ^4^Department of Liver Transplantation, Asian Institute of Gastroenterology, Hyderabad, India; ^5^Department of Gastroenterology, Asian Institute of Gastroenterology, Hyderabad, India

## Abstract

The COVID-19 pandemic has impacted transplantation worldwide in a major way with infections and post-COVID-19 complications contributing to increased mortality and morbidity. We present a case of a 42-year-old lady who developed rhino-orbital mucormycosis in a postliver transplantation period. The initial presentation of the patient was very subtle. She never had overt COVID-19 infection before surgery and in the recovery period. Though cases of mucormycosis in the wound site have been reported, this would be one of the rare cases of rhino-orbital mucormycosis postliver transplantation. This infection leads to a rapid downhill course and the death of the patient. Atypical infections and presentations need to be monitored in postliver transplantation patients who are getting operated on during this pandemic, and a high level of clinical suspicion is required to pick up these cases at an early stage.

## 1. Introduction

Coronavirus disease-2019 (COVID-19) pandemic has affected liver transplantation programmes worldwide and has thrown new challenges in both the pre and posttransplantation periods. Though, currently, many guidelines are in place, yet in the initial phase of pandemic, weighing the risk-benefit ratio of transplantation versus adverse outcome due to SARS-CoV-2 infection was tough [1].

Postliver transplantation outcome has shown varied results during the pandemic. In a systematic review, 23% of posttransplantation patients were found to be infected with severe COVID-19 infection with 55.9% cases requiring modification of immunosuppression [2]. In addition, recent data from the West have shown “excess deaths” in all solid organ transplantations during the pandemic, with the number of deaths mirroring the COVID-19 incidence [3].

In India, during the second wave of the COVID-19 pandemic, rhino-orbital mucormycosis was increasingly reported in diabetic patients (81.2%) and patients with corticosteroid use (79.69%) requiring oxygen supplementation [4]. However, in the published case series, no case was reported in postliver transplantation patients, though they too had a common risk factor of use of corticosteroids. The worst prognosis has been noted in patients with involvement of the brain and those who had a SOFA score of greater than 2 at presentation [5].

We present a case of rhino-orbital mucormycosis in a postliver transplantation patient. The case brings to forefront the subtle onset of the disease and the need of high index of clinical suspicion for diagnosis. Early diagnosis can save the life of many of these patients with good outcome [6].

## 2. Case Report

We present a case of a 42-year-old gentleman who underwent living donor liver transplantation on 25^th^ May 2021 for alcohol use disorder-related decompensated chronic liver disease with MELD score 28. The ABO compatible donor was his wife who had no comorbidities except for hypothyroidism. Both the donor and the recipient were negative for severe acute respiratory syndrome virus 2 (SARS-CoV-2) on reverse transcriptase polymerase chain reaction (RT-PCR) done on two occasions with the last test done 24 hours before surgery. As per protocol of our institute, the COVID-19 antibodies were also tested and found to be negative 24 hours prior to liver transplantation. Neither the recipient nor the donor was vaccinated against SARS-CoV-2.

**Figure 1:**
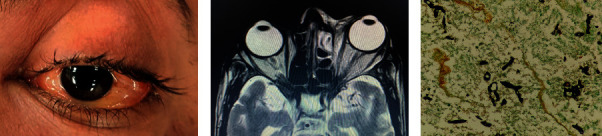
Picture showing lid edema and ptosis of left eye lid (a) with MRI showing mucormycosis deposits in the left paranasal sinuses and orbit (b). Staining with Grocott methenamine silver stain showing septate hyphae of mucormycosis (c).

The postoperative recovery period was uneventful, and the patient was treated with steroids, tacrolimus, and mycophenolate mofetil as per protocol. The dose of prednisolone was tapered to 15 mg/day on the fifteenth day postsurgery. The patient did not have any fever or constitutional symptoms in the postoperative period. However, on posttransplantation day 20, he complained of sudden onset redness of the left periorbital area with pain in the left eye (Figure 1(a)). An immediate in-house ophthalmology consultation was sought. On examination, there was ptosis of the left upper eyelid and mild proptosis of the left eye along with conjunctival redness. Contrast-enhanced magnetic resonance imaging of the orbit, paranasal sinuses, and the brain showed thickening of the medial rectus and superior oblique muscles with fat stranding. In addition, contiguous areas of nonenhancing tissue were noted in the paranasal sinuses suggestive of angioinvasive fungal infection (Figure 1(b)). Qualitative PCR from swabs taken from the left nostril was positive for *Mucorales* deoxyribonucleic acid, which is a sensitive marker for invasive mucormycosis. His repeat test for SARS-CoV-2 RT-PCR and rapid antigen tests were negative. However, his immunoglobulin (Ig) G for COVID-19 was now positive, while IgM was negative. A computerized tomography of the chest showed no evidence of pulmonary COVID-19 infection or any pulmonary mucormycosis.

Nasal swab stain revealed hyphae of mucormycosis on Grocott methenamine silver stain (Figure 1(c)). Based on reports of nasal swab, positive DNA for *Mucorales* and radiographic features [7], a diagnosis of rhino-orbital mucormycosis was made. Systemic antifungals (intravenous liposomal-amphotericin *B*, 10 mg/kg/day, with oral posaconazole 300 mg twice daily on day 1 followed by 300 mg once daily) were initiated. Immunosuppressants tacrolimus and mycophenolate mofetil were stopped. However, even after five days of initiation of antifungals, the patient did not show any clinical signs of improvement. A repeat contrast enhanced MRI of the orbit and paranasal sinuses showed worsening of the lesions with invasion of the orbital bone. Orbital exenteration with the eyelid sparing technique was planned, but surgical consent was refused by the family. Despite continuing conservative management for next 3 days, the patient had a downhill course and succumbed to his infection.

Informed written consent was taken for publication of this case from the patient and also next of kin.

## 3. Discussion

Rhino-orbital mucormycosis is frequently caused by fungal infection due to *Rhizopus* spp., *Mucor* spp., and *Lichtheimia* spp. [8] During the COVID-19 pandemic, there has been an increase in the incidence of such cases [9]. The source of infection is usually from the paranasal sinuses, and majority of the patients presents with unilateral facial edema and proptosis [10]. The European Confederation of Medical Mycology (ECMM) has laid down specific guidelines for diagnosis and includes demonstration of fungal hyphae in microscopy and culture along with molecular methods for direct detection of fungal deoxyribonucleic acid [11].

Steroids use during the pandemic has been implicated as the most common predisposing factor for rhino-orbital mucormycosis [12]. Other coexisting diseases such as diabetes mellitus has been associated with majority of the cases [13]. Longer length of hospital stay in posttransplantation immunosuppressed patients may predispose them to multiple infections including mucormycosis [14]. A rare case report of rhino-orbital maxillary mucormycosis after solid organ transplantation has been reported in the nonpandemic era also [15].

One interesting finding is that despite the use of steroids in posttransplantation patients, the reported incidence of invasive mucormycosis is rare in the nonpandemic era, and therefore, questions have been raised as to whether the SARS-CoV-2 virus itself predisposes patients to mucormycosis.

After solid organ transplantation, abdominal mucormycosis has been reported, and the most common route of infection is contamination during surgery [16]. However, in our case, since the surgical wound was not involved, the chances of contacting the infection from the operation theatre during surgery were very remote. The possibility of SARS-CoV-2 RT-PCR-negative COVID-19 infection was assumed as IgG and IgM which was negative pretransplantation changed to IgG positive posttransplantation. SARS-CoV-2 IgG positivity indicates asymptomatic COVID-19 during the recovery period, which may be masked by immunosuppressants.

Though COVID-19 is showing a downward trend, possibility of a fourth wave still looms large. This case would serve as an index case of rhino-orbital mucormycosis in postliver transplantation patients and make transplant physicians keep their index of suspicion high for any periorbital swelling or ocular pain after transplantation.

## 4. Conclusion

Despite extensive screening of patients in the pretransplantation period, the risk of contracting asymptomatic COVID-19 in the posttransplantation period is possible. However, the exact source and predisposing factor in this rare case will remain an enigma. High index of suspicion is required for any atypical symptom that may occur in the posttransplantation period in this era of COVID-19 pandemic.

## Data Availability

The data used to support this study are available from the corresponding author upon request.

## Abbreviations

SARS-CoV-2: Severe acute respiratory syndrome coronavirus 2
IgG: Immunoglobulin G
RT-PCR: Reverse transcriptase polymerase chain reaction.
